# Peak Expiratory Flow Variability after Abdominal Surgery: A Prospective Observational Study in Peru

**DOI:** 10.2174/0118743064442343260505045704

**Published:** 2026-05-23

**Authors:** Diana P. Reyes-Alata, Milagros N. Rosas-Sudario, Miguel A. Arce-Huamani

**Affiliations:** 1 Universidad Privada Norbert Wiener, Lima, Peru; 2 Programa académico de Medicina Humana, Facultad de Ciencias de la Salud, Universidad Privada Norbert Wiener, Lima, Perú

**Keywords:** Peak expiratory flow rate, Abdominal surgery, Postoperative pulmonary complications, Respiratory function tests, Perioperative care, Pulmonary rehabilitation

## Abstract

**Introduction:**

Peak Expiratory Flow (PEF) is a practical marker of airway patency and cough effectiveness, yet perioperative evidence from Latin American surgical populations is limited. This study aimed to quantify postoperative PEF variability after abdominal surgery and identify associated factors.

**Methods:**

We conducted a prospective observational study at a Peruvian hospital in 2024. Adults undergoing elective abdominal surgery performed PEF testing with a Mini-Wright meter preoperatively and on postoperative day 7 using a standard three-maneuver protocol. The highest valid value was retained. The primary outcome was PEF variability, defined as the difference between preoperative and postoperative PEF (L/min). Group comparisons used non-parametric tests, and multivariable linear regression identified independent predictors.

**Results:**

Eighty-two patients were included; 58.5% were women, and the median age was 48 years. Mean preoperative and postoperative PEF values were 367.9 ± 71.9 and 346.0 ± 74.8 L/min, respectively. Median PEF variability was 20 L/min (IQR 10–30). Variability did not differ by sex or BMI. Age was the only independent predictor of greater decline.

**Discussion:**

Abdominal surgery was associated with an early postoperative reduction in expiratory flow, particularly in older adults.

**Conclusion:**

Routine perioperative PEF assessment may help identify patients who require closer respiratory monitoring and early supportive therapy in resource-constrained surgical settings.

## 1 INTRODUCTION

Peak Expiratory Flow (PEF) is recognized as a practical indicator for assessing respiratory function, commonly used to evaluate airway patency, respiratory muscle strength, and the capacity for effective cough [1]. It is a simple, low-cost bedside measure that can be applied in a wide range of clinical settings, including perioperative care [2]. In surgical populations, impaired expiratory flow and ineffective cough are clinically relevant because they may compromise airway clearance and contribute to Postoperative Pulmonary Complications (PPCs), particularly pneumonia, atelectasis, and respiratory failure [3, 4]. Current evidence suggests that reduced PEF is associated with poorer airway-clearance capacity and adverse respiratory outcomes in selected surgical and high-risk clinical populations [4, 5].

Current evidence indicates that low PEF rates can predict the development of pneumonia and other respiratory complications; for instance, in a retrospective cohort of 513 patients undergoing esophagectomy, a PEF below 80% significantly correlated with higher postoperative pneumonia incidence [4]. Similarly, among older adults, lower PEF values have been directly linked with increased risks of Motoric Cognitive Risk syndrome (MCR), emphasizing the broader implications of impaired pulmonary function on overall health and morbidity [5]. Furthermore, a significant 10-year cohort study in Eastern China found that reduced PEF was independently associated with elevated risks of cardiovascular diseases, all-cause mortality, and cardiovascular-related mortality, underscoring the importance of PEF beyond respiratory conditions [6].

However, despite the recognized clinical value of PEF, important uncertainties remain regarding its perioperative use. Measurement quality depends on standardized technique, patient coaching, and reproducibility criteria, all of which may influence the reliability of effort-dependent flow measures [2]. In addition, although preoperative PEF has shown prognostic value for postoperative respiratory outcomes in some surgical settings, evidence remains limited for abdominal surgery, particularly in Latin American populations [4, 7, 8]. Therefore, more context-specific perioperative evidence is still needed.

Given these knowledge gaps and methodological concerns, a more comprehensive understanding of PEF variability and its predictive accuracy for postoperative respiratory complications in specific surgical populations, such as those undergoing abdominal surgery, is necessary.

Therefore, the objective of the present study was to determine the variability of peak expiratory flow in adult patients undergoing abdominal surgery at a Peruvian hospital.

## 2 MATERIALS AND METHODS

### 2.1 Study Design and Setting

We conducted a prospective observational study at a secondary-care hospital in Chorrillos, Lima, Peru (February–September 2024). The primary aim was to quantify postoperative variability in Peak Expiratory Flow (PEF) after abdominal surgery and to explore associated factors. The study captured standardized pre- and postoperative measurements to enable within-patient comparisons over time.

### 2.2 Population and Sample

Adults (18–80 years) scheduled for elective abdominal surgery were consecutively enrolled. Inclusion criteria were: ability to follow verbal instructions and provision of written informed consent. Exclusion criteria were: emergency procedures; acute or chronic respiratory disease; inability to perform valid PEF maneuvers; or failure to complete the study protocol. Consecutive sampling minimized selection bias within the target surgical population.

We aimed to enroll approximately 80 adults to estimate the within-patient change in Peak Expiratory Flow (PEF) with acceptable precision and adequate power. Specifically, the target was a two-sided 95% CI half-width of ~15 L/min for the mean paired change and ≥ 80% power (α = 0.05) to detect a clinically relevant mean decline of ≥ 20 L/min, assuming a conservative within-patient SD of ~60 L/min. We ultimately included 82 participants, meeting this a *priori* target.

### 2.3 Outcomes and Measurements

The primary outcome was PEF variability, defined as the difference between preoperative and postoperative PEF (L/min).

**PEF protocol:** Using a Mini-Wright Peak Flow Meter, participants performed three forced expiratory maneuvers at each time point (preoperative and postoperative day 7). Postoperative day 7 was selected because it coincides with the routine first postoperative ward or outpatient review at this hospital and ensures that most patients are clinically stable enough to perform reproducible forced expiratory efforts. The highest reading was retained, provided it did not differ by more than 20 L/min from the next highest value. A single Mini-Wright device was used for all measurements; before each session, the research team visually inspected the meter, verified that the pointer moved freely and returned to zero, and replaced the disposable mouthpiece, following the manufacturer’s recommendations. No additional laboratory calibration was undertaken.

**Perioperative clinical data:** Analgesia regimens and postoperative pain scores were managed according to standard institutional protocols and were not prospectively standardized or recorded for research purposes. Postoperative respiratory symptoms (such as cough, dyspnoea, or wheeze) were monitored as part of routine clinical care but were not systematically captured in the study database and therefore were not included as covariates in the analyses.

**Patient characteristics:** Sex (male/female), age groups (18–30, 31–40, 41–50, 51–60, 61–80 years), weight, height, and Body Mass Index (BMI). BMI was computed as kg/m^2^ and categorized as underweight/normal, overweight, or obesity per WHO conventions.

### 2.4 Statistical Analysis

Analyses were conducted in Stata v18. We first summarized the sample using frequencies (%) for categorical variables and means ± Standard Deviation (SD) or medians (interquartile range, IQR) for continuous variables, as appropriate. Distributional assumptions were assessed with the Kolmogorov–Smirnov test. Because PEF variability was non-normally distributed, we used non-parametric tests for group comparisons: Wilcoxon signed-rank test (pre- *vs.* postoperative PEF), Mann–Whitney U test (sex), and Kruskal–Wallis test (age groups and BMI categories). We then fitted a single multivariable linear regression model to identify independent predictors of PEF variability. Age group, sex, and BMI category were entered simultaneously based on their a *priori* clinical relevance to expiratory performance; no automated variable-selection procedures were applied in order to avoid overfitting in this modest sample. We examined standard assumptions (linearity, homoscedasticity, independence, and normality of residuals) using residual-versus-fitted plots, normal probability plots, and leverage diagnostics, and we did not identify patterns suggesting serious violations or influential outliers. A two-sided *p*-value < 0.05 was considered statistically significant, and 95% Confidence Intervals (CI) and *p*-values are reported for all model coefficients.

## 3 RESULTS

We analyzed 82 adults undergoing abdominal surgery (58.5% women). The largest age group was 61–90 years (30.5%), followed by 18–30 (22.0%), 41–50 (20.7%), 31–40 (14.6%), and 51–60 (12.2%). Most patients were overweight (57.3%), with 31.7% classified as obese and 11.0% as normal/underweight. Median age was 48 years (IQR 33–68). Mean preoperative PEF was 367.9 ± 71.9 L/min, and mean postoperative PEF was 346.0 ± 74.8 L/min. Median PEF variability (preoperative minus postoperative) was 20 L/min (IQR 10–30) (Table **1**).

Sex-based differences in PEF variability were not significant (median 20 *vs* 20 L/min; *p*-value = 0.83). Variability increased with age across groups (*p*-value = 0.003), with medians ranging from 10 L/min in 18–30 years to 30 L/min in 51–60 years. There were no differences by BMI category (*p*-value = 0.86) (Table **2**).

In multivariable linear regression, age group remained the only independent predictor of higher PEF variability. Compared with 18–30 years, adjusted β coefficients (95% CI) were 17.50 (5.07, 29.93) for 41–50 years (*p*-value = 0.01), 21.50 (7.74, 35.27) for 51–60 years (*p*-value = 0.003), and 20.87 (10.19, 31.61) for 61–90 years (*p*-value < 0.001); sex and BMI were not significant (Table **3**).

**Fig. (1):**
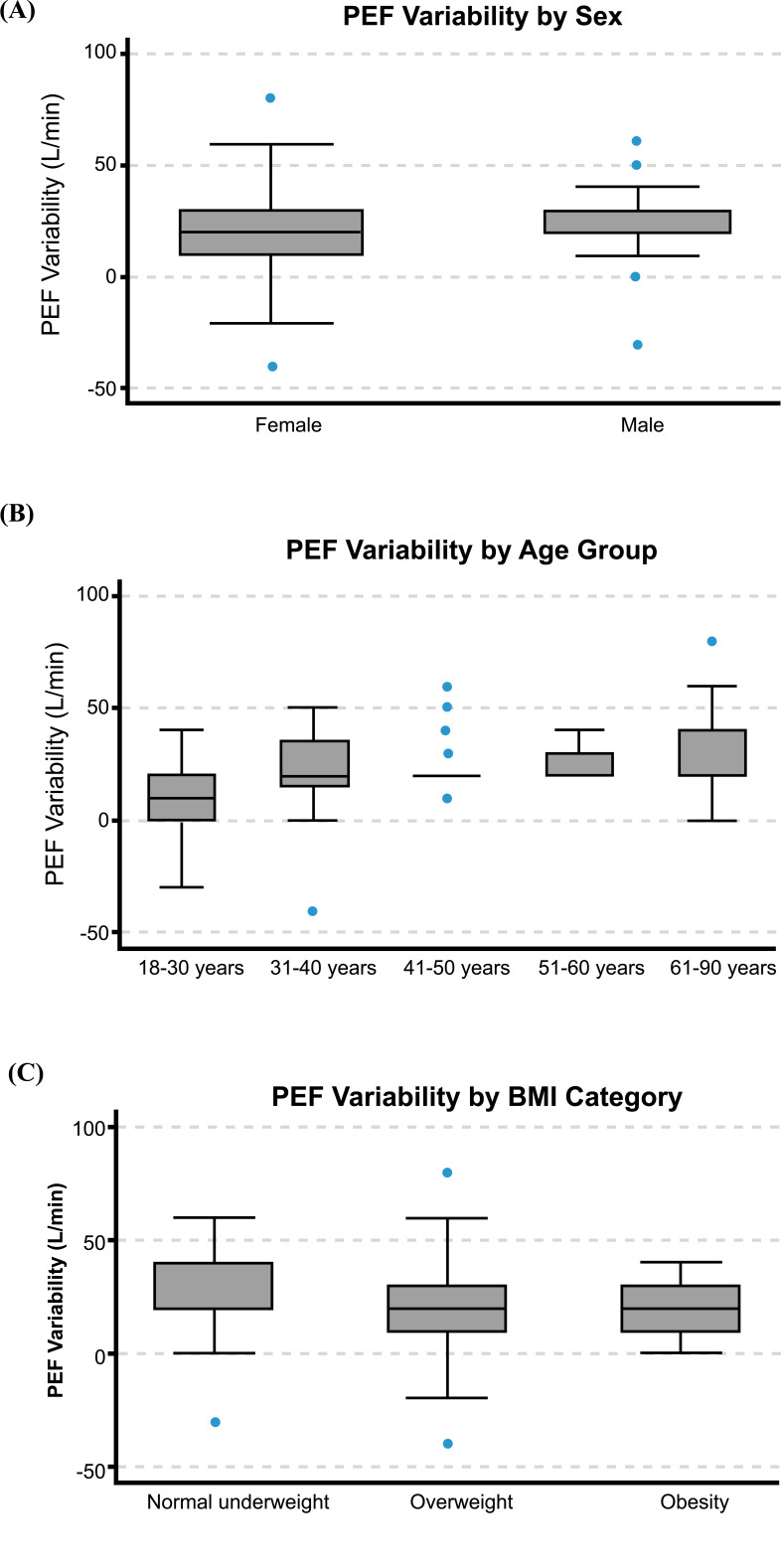
Distribution of postoperative PEF variability by sex (**A**), age group (**B**), and BMI category (**C**). Each boxplot shows the median, Interquartile Range (IQR), and outliers for PEF variability (L/min). No significant differences were found by sex or BMI category, whereas a significant difference was observed across age groups (*p*-value = 0.003, Kruskal–Wallis test).

The boxplots illustrate these patterns, showing the median, interquartile range, and individual points identified as outliers by the boxplot algorithm: no difference by sex or BMI, and a clear age-related gradient (Kruskal–Wallis *p*-value = 0.003) (Fig. **1**). All data points, including those plotted as outliers, were retained in the statistical analyses.

## 4 DISCUSSION

This prospective observational study shows that postoperative Peak Expiratory Flow (PEF) variability operationalized as the absolute decline between preoperative and day-7 values has a median of 20 L/min after abdominal surgery, and that age is the only independent determinant of a larger decline. Sex and BMI were not associated with PEF variability. These findings underscore a simple, low-cost functional marker that captures early postoperative respiratory compromise and can inform targeted respiratory therapy in routine care.

Our results align with prior studies reporting postoperative reductions in expiratory performance across surgical populations. Rossi *et al*. (2024) documented a marked fall in PEF after bariatric surgery with only partial recovery at discharge [9]. Shah *et al*. (2020) observed large early postoperative drops after elective laparotomy, with incomplete recovery by day 7 [10]. Misquith *et al*. (2016) similarly showed the steepest PEF deficits within 24–48 hours after upper abdominal surgery, with persistent reductions thereafter [11]. Together, these data across different procedures and settings support a consistent postoperative pattern likely driven by pain-related splinting, diminished diaphragmatic excursion, and blunted deep breathing.

The pronounced age gradient observed in this study is biologically plausible and consistent with recent literature. Sun *et al*. (2025) reported age-related declines in PEF/PEFR and respiratory muscle indices, with advanced age predicting worse outcomes [12]. Chang *et al*. (2021) also linked older age to lower preoperative PEF and higher postoperative pulmonary risk in oncologic surgery [8], and Liu *et al*. (2021) found that older patients were more likely to exhibit ineffective cough after thoracic procedures [7]. Age-related sarcopenia, reduced chest-wall compliance, impaired cough mechanics, and broader geriatric frailty likely converge to lower PEF, rendering older adults particularly vulnerable to mucus retention, atelectasis, and clinically significant postoperative respiratory decline.

Taken together, these age-related changes mean that older adults often start the perioperative course with lower expiratory reserve. Even when they are able to perform three technically acceptable maneuvers, pain, sedation, and fatigue may further limit their capacity to take a full inspiration and generate an explosive exhalation, amplifying the postoperative decline in PEF. Thus, a similar absolute drop in flow may represent a far greater proportional loss of reserve in an older, frail individual than in a younger counterpart.

These observations also support the rationale for targeted prehabilitation in high-risk older adults. Multicomponent prehabilitation programs that combine aerobic conditioning, respiratory muscle training, and functional strength exercises have been associated with improved postoperative functional capacity and fewer complications in selected surgical populations. Although we did not implement or evaluate prehabilitation in this cohort, our findings suggest that older adults with low baseline PEF or anticipated larger declines could be priority candidates for short, structured preoperative interventions aimed at augmenting cough effectiveness and ventilatory reserve.

Although we did not adjudicate postoperative pulmonary complications in this study, the direction and magnitude of the observed PEF decline are clinically meaningful. Prior work has shown that lower perioperative PEF values and relative decrements from baseline are associated with higher risks of pneumonia and other postoperative pulmonary complications in thoracic and abdominal surgery [3, 5, 7, 8]. In our cohort, the median absolute decline of 20 L/min at day 7 suggests at least a modest deterioration in expiratory flow, particularly among older adults, that could plausibly translate into greater vulnerability to mucus retention, atelectasis, and infection. Future longitudinal studies that combine serial PEF trajectories with systematically adjudicated PPCs are needed to define robust risk thresholds and to clarify how much change in PEF should trigger intensification of respiratory therapy.

By contrast, we found no association of sex or BMI with postoperative PEF variability in this cohort. One possible explanation is that early postoperative changes in expiratory flow may depend more on perioperative factors that directly affect respiratory effort and airway clearance, such as pain control, breathing coaching, early mobili-zation, and adherence to respiratory therapy, than on anthropometric characteristics alone. Anthropometrics alone may fail to capture perioperative determinants more proximal to expiratory flow, such as pain control, coached breathing, early mobilization, and adherence to airway-clearance exercises.

From a practical perspective, serial PEF monitoring could be integrated as a simple screening tool to identify patients who might benefit from early or intensified respiratory therapy. Individuals showing larger-than-expected declines in PEF could be triaged to more frequent physiotherapy sessions, coached in deep breathing, or device-based airway clearance, while those with stable flows follow standard postoperative pathways. Compared with spirometric indices such as Forced Vital Capacity (FVC) or formal assessments of incentive spirometry performance, PEF meters are inexpensive, portable, and easy to use on busy surgical wards, particularly in resource-limited settings. Rather than replacing these more comprehensive measures, PEF could complement them by providing a rapid bedside signal of deteriorating expiratory performance and helping clinicians prioritize respiratory care for those at greatest risk.

Strengths include a prospective design; a standardized three-maneuver PEF protocol with validity criteria; and within-patient comparisons that reduce between-subject variability. Limitations include the single-center setting and modest sample size, which constrain precision for subgroup analyses and limit generalisability beyond similar secondary-care hospitals; the absence of pain or analgesia metrics that likely influence expiratory effort; and the lack of adjudicated Postoperative Pulmonary Complications (PPCs) or earlier time points (days 1–3). Future studies should map PEF trajectories in the immediate postoperative period and link thresholds with PPCs and length-of-stay to define actionable cut-offs.

Embedding pre- and postoperative PEF checks into perioperative pathways can flag patients, especially older adults, who warrant early, RT-led, age-attentive respira-tory therapy (coached deep-breathing and coughing, incentive spirometry, airway-clearance techniques, early mobilization, and optimized analgesia). Because PEF meters are inexpensive and easy to deploy at the bedside, this strategy is feasible in resource-constrained settings and scalable across similar Latin American hospitals. Given these design features, our findings should be extrapolated cautiously to other Latin American surgical populations with different case-mix, perioperative pathways, or resource levels.

## CONCLUSION

Postoperative Peak Expiratory Flow (PEF) declines after abdominal surgery, and the reduction is disproportionately larger in older adults. Integrating routine pre- and postoperative PEF checks into periope-rative pathways can promptly identify patients who warrant early, age-attentive, RT-led respiratory therapy (coached deep-breathing and coughing, incentive spiro-metry, airway-clearance techniques, early mobilization, and optimized analgesia). This pragmatic, low-cost approach is feasible in resource-constrained settings and may help reduce postoperative pulmonary risk.

## Figures and Tables

**Table 1:** Sociodemographic, clinical, and functional characteristics of the study population.

Characteristics	n (%)
**Sex**	
Female	48 (58.54)
Male	34 (41.46)
**Age group (years)**	
18–30 years	18 (21.95)
31–40 years	12 (14.63)
41–50 years	17 (20.73)
51–60 years	10 (12.20)
61–90 years	25 (30.49)
**Body mass index (BMI)**	
Normal/underweight	9 (10.98)
Overweight	47 (57.32)
Obesity	26 (31.71)
**Quantitative variables**	
Age*	48 (33–68)
Preoperative PEF**	367.9 ± 71.9
Postoperative PEF**	346.0 ± 74.8
Variability*	20 (10–30)

**Note:** Data are n (%) unless otherwise indicated. Age and PEF variability are median (IQR); pre- and postoperative PEF are mean ± SD. **Abbreviations:** PEF, peak expiratory flow; BMI, body mass index; SD, standard deviation; IQR, interquartile range.

**Table 2:** Peak Expiratory Flow (PEF) variability by sex, age group, and BMI category.

Group	n	Median variability (IQR)	*p*-value
**Sex**	-	-	0.83^1^
Female	48	20 (10–30)	-
Male	34	20 (20–30)	-
**Age group**	-	-	0.003^2^
18–30 years	18	10 (0–20)	-
31–40 years	12	20 (15–35)	-
41–50 years	17	20 (10–30)	-
51–60 years	10	30 (20–40)	-
61–90 years	25	20 (10–40)	-
**BMI**	-	-	0.86^2^
Normal/underweight	9	20 (20–40)	-
Overweight	47	20 (10–30)	-
Obesity	26	20 (10–30)	-

**Note:** Statistical tests: ^1^ Mann–Whitney U test; ^2^ Kruskal–Wallis test. **Abbreviations:** PEF, peak expiratory flow; BMI, body mass index; IQR, interquartile range.

**Table 3:** Crude and adjusted predictors of PEF variability: multivariable linear regression.

Variable	Crude β (95% CI)	*p*-value	Adjusted β (95% CI)	*p*-value
**Sex**	-	-	-	-
Female	Reference	-	Reference	-
Male	-1.32 (–9.47, 6.82)	0.75	-4.02 (–11.83, 3.80)	0.31
**Age group**	-	-	-	-
18–30 years	Reference	-	Reference	-
31–40 years	11.94 (–0.64, 24.53)	0.06	12.17 (–0.49, 24.83)	0.06
41–50 years	14.65 (3.22, 26.09)	0.01	17.50 (5.07, 29.93)	0.01
51–60 years	19.11 (5.79, 32.43)	0.006	21.50 (7.74, 35.27)	0.003
61–90 years	19.51 (9.07, 29.92)	<0.001	20.87 (10.19, 31.61)	<0.001
**BMI**	-	-	-	-
Normal/underweight	Reference	-	Reference	-
Overweight	0.33 (–12.97, 13.63)	0.96	-3.53 (–16.47, 9.42)	0.59
Obesity	-1.45 (–15.59, 12.68)	0.84	-7.44 (–20.99, 6.11)	0.28

**Note:** Values are β coefficients (95% CI). Crude estimates are from univariable models; adjusted estimates are from multivariable models including all variables. **Abbreviations:** CI, confidence interval; BMI, body mass index; PEF, peak expiratory flow.

## Data Availability

The data supporting the findings of this study were derived from the de-identified study database generated by the authors from standardized peak expiratory flow measurements and perioperative clinical records. The dataset is not publicly available because it contains potentially identifiable clinical information from human participants. However, de-identified data may be made available from the corresponding author upon reasonable request and subject to approval by the Institutional Research Ethics Committee of Universidad Privada Norbert Wiener.

## LIST OF ABBREVIATIONS

BMI: Body Mass Index
CI: Confidence Interval
IQR: Interquartile Range
PEF: Peak Expiratory Flow
PPCs: Postoperative Pulmonary Complications
SD: Standard Deviation

## STANDARDS OF REPORTING

STROBE guidelines were followed.
